# Impact of *Lactocaseibacillus* (*Lactobacillus*) *paracasei* sup. *paracasei* TISTR 2593 Probiotic Supplementation on the Gut Microbiome of Hypercholesterolemia Patients: A Randomized Controlled Trial

**DOI:** 10.3390/nu16172916

**Published:** 2024-09-01

**Authors:** Kamonsri Nuankham, Jaruwan Sitdhipol, Pennapa Chonpathompikunlert, Jurairat Khongrum, Romteera Kittichaiworakul, Pitiporn Noisagul, Patcharawadee Thongkumkoon, Tanyaluck Kampoun, Sivamoke Dissook

**Affiliations:** 1 Biodiversity Research Centre (BRC), Thailand Institute of Scientific and Technological Research (TISTR), Pathum Thani 12120, Thailand; 2 Master of Biomedical Data Science, Faculty of Science, Kasetsart University, Bangkok 10900, Thailand; 3 Multidisciplinary Research Institute, Chiang Mai University, Chiang Mai 50200, Thailand; 4 Department of Biochemistry, Faculty of Medicine, Chiang Mai University, Chiang Mai 50200, Thailand; 5 Center of Multidisciplinary Technology for Advanced Medicine (CMUTEAM), Faculty of Medicine, Chiang Mai University, Chiang Mai 50200, Thailand; 6 Office of Research Administration, Chiang Mai University, Chiang Mai 50200, Thailand

**Keywords:** *Lactocaseibacillus* (*Lactobacillus) paracasei* sup. *paracasei* TISTR 2593, gut microbiome, probiotics, hypercholesterolemia, metabolic health

## Abstract

Probiotics have shown potential in managing hypercholesterolemia and related metabolic conditions. This study evaluated the effects of *Lactocaseibacillus* (*Lactobacillus) paracasei* sup. *paracasei* TISTR 2593 on the gut microbiome and metabolic health in patients with hypercholesterolemia, and was registered in the Thai Clinical Trial Registry (TCTR 20220917002). In a randomized, double-blind, placebo-controlled trial, 22 hypercholesterolemic participants received either the probiotic or a placebo daily for 90 days. Fecal samples collected before and after the intervention revealed significant microbiome changes, including a decrease in *Subdoligranulum*, linked to rheumatoid arthritis, and an increase in *Flavonifractor*, known for its anti-inflammatory properties. Additionally, the probiotic group exhibited a significant reduction in low-density lipoprotein cholesterol (LDL-C) levels. These findings suggest that *L. paracasei* TISTR 2593 can modulate the gut microbiome and improve metabolic health, warranting further investigation into its mechanisms and long-term benefits.

## 1. Introduction

In recent years, the food industry has faced intensified competition, coupled with a significant shift in consumer behavior towards health-oriented products. Among these, probiotics have emerged as a highly favored category over the past two decades. Probiotics are live microorganisms that, when administered in adequate amounts, confer health benefits to the host. They are commonly found in foods such as yogurt, kefir, kimchi, and miso [1,2,3].

The concept of probiotics stems from the understanding that these beneficial bacteria play a crucial role in maintaining and restoring gut flora, thereby enhancing gastrointestinal health. Probiotics such as *Lactobacillus acidophilus* [4], *Lactobacillus helveticus* [5], *Lactobacillus casei* [6], *Lactobacillus bulgaricus* [7], *Lactobacillus reuteri* [8], and *Lactobacillus GG* [9], along with *Bifidobacterium bifidum* [10], have been extensively studied for their health benefits. These strains are known for their ability to withstand the acidic and alkaline conditions of the gastrointestinal tract, adhere to the intestinal epithelium, and inhibit pathogenic bacteria through the production of antimicrobial substances.

*Lactobacillus paracasei* subsp. *paracasei*, initially classified by Collins et al. in 1989 [11], has been widely studied for its beneficial effects on lipid metabolism. In 2020, the genus was renamed to *Lacticaseibacillus paracasei* subsp. *paracasei* by Zheng et al. due to advancements in bacterial taxonomy [12]. This reclassification reflects the evolving understanding of the phylogenetic relationships within the *Lactobacillaceae* family. The strain *Lacticaseibacillus paracasei* TISTR 2593 used in this study has shown promise in previous research for its potential to lower LDL-C levels and improve gut health. Our previous studies have documented the efficacy of probiotics in improving various health parameters, including lipid metabolism and immune response. Specifically, a randomized double-blind placebo-controlled trial demonstrated that supplementation with *L. paracasei* TISTR 2593 significantly improved cholesterol metabolism and atherosclerosis-related parameters in subjects with hypercholesterolemia [13].

This study extends the findings of our previously published work [13] by incorporating a detailed microbiome analysis. While the initial study focused on the effects of *L. paracasei* TISTR 2593 on lipid metabolism and atherosclerosis-related parameters, the current investigation explores the probiotic’s impact on gut microbiome composition and diversity. Our findings reveal significant shifts in microbial taxa, which correlate with improved metabolic outcomes, providing a comprehensive understanding of the probiotic’s multifaceted benefits.

## 2. Materials and Methods

### 2.1. Subjects

This study adhered to the principles outlined in the Declaration of Helsinki. Approval for the research protocol was obtained from the Ethical Committee of the Human Experimentation Committee, Research Institute for Health Science (RIHES) at Chiang Mai University, Thailand (Project No. 11/64) and was registered in the Thai Clinical Trials Registry (TCTR) (number TCTR20220917002). Informed consent was obtained from all participants prior to their inclusion in the study. This study was designed as a pilot study aimed at exploring the potential effects of *L. paracasei* TISTR 2593 on the gut microbiome in patients with hypercholesterolemia. Given the exploratory nature of this research, we did not conduct a new power calculation for this subset of participants. Instead, the sample size was determined based on the availability of samples from a previous study, ensuring that there was adequate representation from both the probiotic and placebo groups. The inclusion criteria included male and female participants (non-pregnant), aged 30–65 years, with mild to moderate hypercholesterolemia, defined by serum LDL-C levels between 100 and 159 mg/dL, and a body mass index (BMI) between 19 and 30 kg/m^2^. Exclusion criteria included a history of cardiovascular disease, secondary dyslipidemia, diabetes, severe hypertension, or any changes in exercise routines, eating habits, or dietary supplements during the study period. The detailed subject recruitment was published in our previous study [13].

### 2.2. Study Design and Treatment

**Figure 1:**
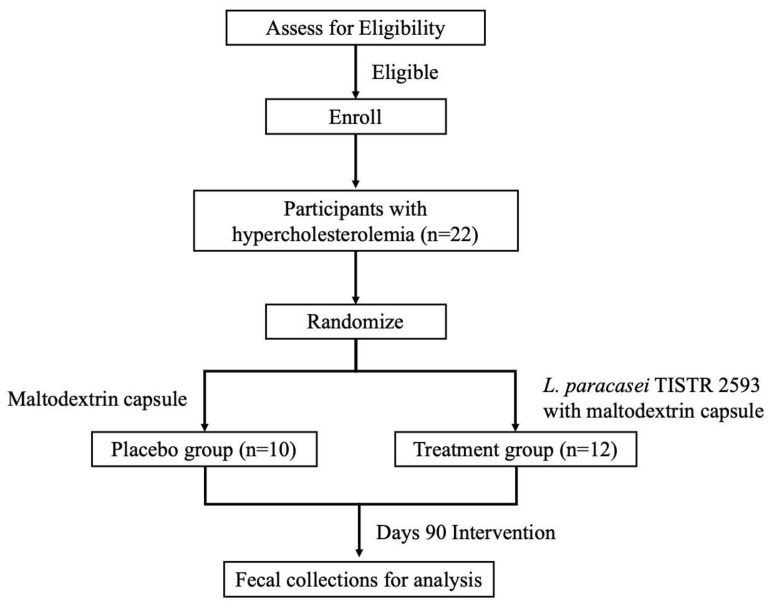
Study design and participant flowchart illustrating the study design and participant allocation. A total of 22 participants with hypercholesterolemia were enrolled and randomized into two groups. The placebo group (*n* = 10) received a daily maltodextrin capsule, while the treatment group (*n* = 12) received a daily capsule containing *L. paracasei* TISTR 2593 with maltodextrin. Both groups underwent a 90-day intervention period, after which fecal samples were collected for microbiome analysis.

The experiment was conducted as described in Figure 1, and the full details could be found in our previous publication [13]. Briefly, we carried out a single-center, prospective, randomized, double-blind, placebo-controlled, parallel-group study. Both the subjects and the investigators were blinded to the treatments administered. The *L. paracasei* TISTR 2593 strain used in this study was freeze-dried and encapsulated in maltodextrin, which served as an excipient. Participants were randomly assigned to one of two groups. The treatment group received *L. paracasei* TISTR 2593 encapsulated in maltodextrin, with each capsule containing 1.05 × 10^9^ CFU/g (350 mg per capsule). Participants in the treatment group were instructed to take three capsules per day, bringing the total daily dosage to 1.05 × 10^9^ CFU. The placebo group received maltodextrin capsules without the probiotic.

Randomization was performed using block randomization, and both the participants and investigators were blinded to the group assignments. Blood samples were collected to assess lipid profiles, including total cholesterol (TC), triglycerides (TG), LDL-C, and high-density lipoprotein cholesterol (HDL-C), as well as fasting blood glucose (FBG) and other relevant metrics, before the intervention and at 45-day and 90-day intervals during the intervention. Fecal samples from both groups were collected at baseline and after 90 days of the intervention.

### 2.3. Feces Collection

Participants were provided with detailed instructions and fecal collection tubes prefilled with DNA/RNA Shield™ (Zymo Research, Irvine, CA, USA). They were instructed to use these tubes for 1–2 days prior to sample collection. Fecal samples were collected using a swab and then placed individually in the fecal collection tubes. These tubes were stored at 4 °C until transported to the laboratory. Upon arrival at the laboratory, the samples were stored at −80 °C until further processing.

### 2.4. DNA Extraction and Sequencing

#### 2.4.1. DNA Extraction

The DNA was extracted using the ZymoBIOMICS^®^-96 MagBead DNA Kit (Zymo Research, Irvine, CA, USA), following the manufacturer’s instructions. The elution volume for the extracted DNA was 50 µL.

#### 2.4.2. Library Preparation and Sequencing

The extracted DNA was prepared for sequencing using the Quick-16S™ NGS Library Prep Kit (Zymo Research, Irvine, CA, USA), targeting the V3-V4 hypervariable region of the 16S rRNA gene. The primers used for amplification were the Quick-16S™ Primer Set V3-V4 (Zymo Research, Irvine, CA, USA). The library preparation process involved PCR reactions conducted in real-time PCR machines to control cycles and minimize PCR chimera formation. The PCR products were quantified using qPCR fluorescence readings and pooled based on equal molarity. The pooled library was cleaned using the Select-a-Size DNA Clean & Concentrator™ (Zymo Research, Irvine, CA, USA), and quantified with TapeStation^®^ (Agilent Technologies, Santa Clara, CA, USA) and Qubit^®^ (Thermo Fisher Scientific, Waltham, WA, USA). The final library was sequenced on an Illumina^®^ MiSeq™ (San Diego, CA, USA) platform using a v3 reagent kit (600 cycles) with a 10% PhiX spike-in for sequencing quality control.

### 2.5. Microbiome Analysis

The sequencing results derived from the amplicon-based sequencing were quality-checked by FastQC software version 0.11.9. Quantitative Insights Into Microbial Ecology 2 (QIIME2; version 2021.8.0) [14] was used to process and categorize the sequence data into the taxonomic abundance data with the SILVA 16S rRNA gene reference database [15] release 138.

### 2.6. Diversity Analysis of the Fecal Microbiome

The taxonomic abundance data were statistically analyzed and visualized through the R software version 4.3.2. The Shannon diversity index was calculated using the phyloseq package [16] and compared between the samples within the group and between the groups. The Orthogonal Partial Least Squares Discriminant Analysis (OPLS-DA) was performed using the rolps package for sample categorization and microbial marker identification. The microbe with a vip value > 1 was assumed to be a distinguishing marker between the categorized samples generated by OPLS-DA. The microbe markers derived from the OLPS-DA were confirmed by comparison of their relative abundance between the categorized sample groups. The ggplot2 package was used to generate the Shannon diversity index box plot, the stack bar graph, and the box plot of the relative abundance.

### 2.7. Statistical Comparison Analysis

All the statistical comparisons were tested using the Wilcoxon rank sum and signed rank tests at a 95% coefficient, which in a paired or unpaired test was defined for within-the-group or between-group comparisons, respectively. Blood parameter statistics were recalculated using data from our previous publication [13]. The ggpubr package was used along with the ggplot2 package to add a *p*-value to the plots.

## 3. Results

**Figure 2:**
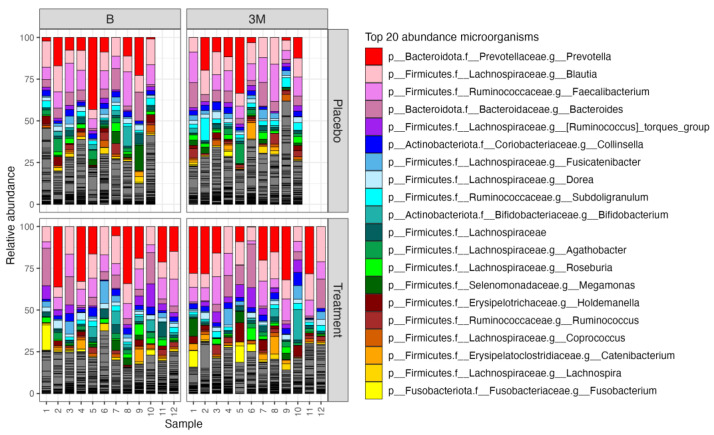
Taxa bar plot illustrating the impact of *L. paracasei* TISTR 2593 supplementation on gut microbiome composition in hypercholesterolemia patients. Relative abundance of the top 20 bacterial taxa in stool samples from patients in the placebo (upper panels) and treatment groups (lower panels) at baseline (B) and after 90 days (3M) of supplementation. Each bar represents the microbial composition of an individual sample.

The impact of *L. paracasei* TISTR 2593 on the gut microbiome of patients with hypercholesterolemic participants was evaluated through a randomized controlled trial. The key findings were significant alterations in the gut microbiome diversity and composition and are depicted in Figure 2.

### Microbiome Profile

**Figure 3:**
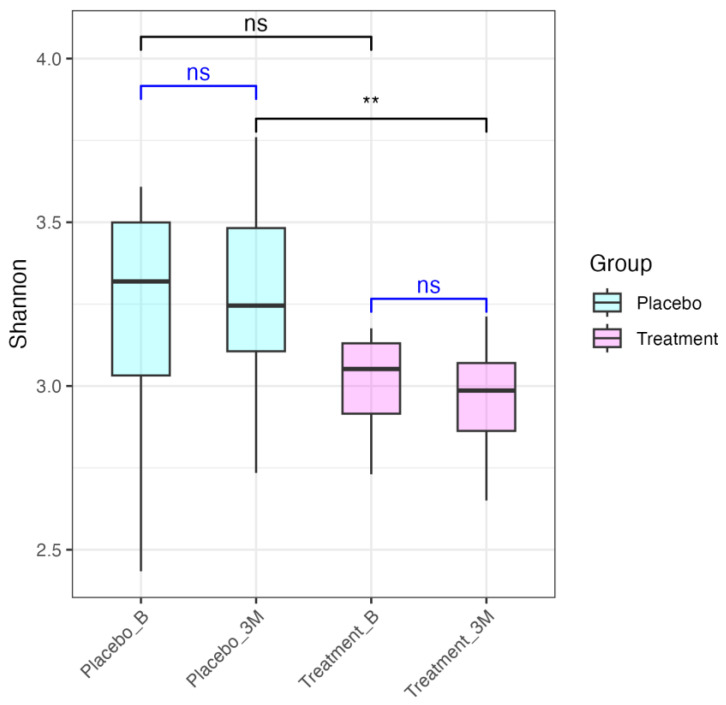
Comparison of Shannon diversity index between the treatment and placebo groups at baseline and after 90 days of intervention. The box plots represent the Shannon diversity index of gut microbiota in the placebo and treatment groups at baseline (Placebo_B and Treatment_B) and after 90 days (Placebo_3M and Treatment_3M). The blue lines represent paired Wilcoxon signed-rank tests comparing baseline and 3-month data within the same group. The black lines indicate the Wilcoxon rank-sum test comparing groups at the same time point. ‘ns’ denotes non-significant differences, while ‘**’ indicates statistically significant differences (*p* < 0.01).

The impact of *L. paracasei* TISTR 2593 supplementation on gut microbiome diversity was assessed using the Shannon diversity index (Figure 3). At baseline, the Wilcoxon test *p*-values showed no significant difference in Shannon diversity index between the placebo and treatment groups (*p* = 0.14). However, after 90 days of supplementation, a significant decrease in microbial diversity was observed in the treatment group compared to the placebo group (*p* = 0.007). Specifically, Figure 3 presents box plots comparing the Shannon diversity index within the placebo and treatment groups at baseline (B) and after 90 days (3M) of supplementation. No significant differences in diversity were observed within either group over the 90 days (placebo, *p* = 0.92; treatment, *p* = 0.57). Figure 3 further highlights the differences in Shannon diversity index between the placebo and treatment groups at baseline and after 90 days. The treatment group exhibited a significant decrease in diversity after 90 days compared to the placebo group, indicating that *L. paracasei* TISTR 2593 supplementation leads to decreased gut microbiome diversity in hypercholesterolemia patients over time. These results suggest that the probiotic intervention selectively modulates the gut microbiome, potentially enhancing the growth of specific taxa without affecting overall microbial composition, which may have implications for gut health and metabolic outcomes in patients with hypercholesterolemia.

**Figure 4:**
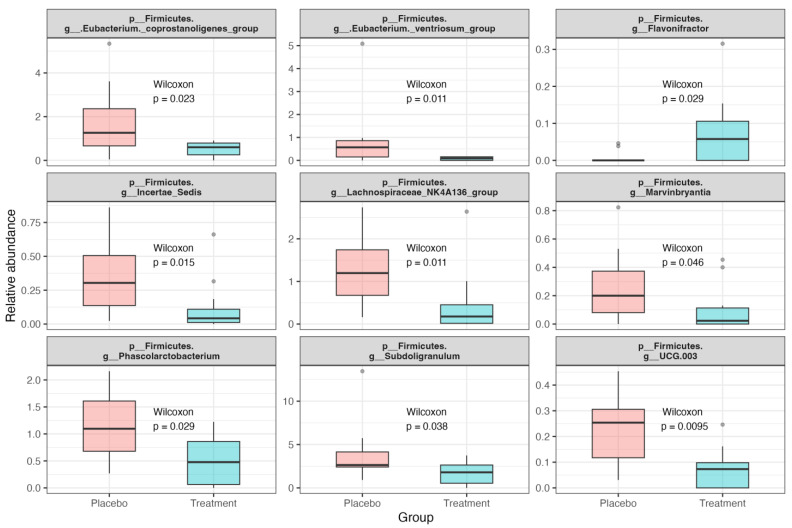
Differential abundance of bacterial taxa in the gut microbiome of hypercholesterolemia patients following *L. paracasei* TISTR 2593 supplementation. Box plots depict the relative abundance of specific bacterial taxa in the placebo and treatment groups after 90 days of supplementation. Each plot represents a different bacterial taxon, showing significant differences in abundance between the placebo and treatment groups. The differences were assessed using the Wilcoxon paired test. The gray dots represent individual data points that are considered outliers, which fall outside the interquartile range of the dataset.

The impact of *L. paracasei* TISTR 2593 supplementation on the relative abundance of various bacterial taxa in the gut microbiome was assessed. The differences were assessed using the Wilcoxon paired test (Figure 4). The data indicate significant differences in the abundance of several bacterial taxa between the placebo and treatment groups after 90 days of supplementation. Specifically, the treatment group exhibited a significant decrease in the relative abundance of *Subdoligranulum* (*p* = 0.037743), *Eubacterium coprostanoligenes* group (*p* = 0.022760), *Lachnospiraceae NK4A136* group (*p* = 0.011038), *Eubacterium ventriosum* group (*p* = 0.010655), *Phascolarctobacterium* (*p* = 0.029329), UCG-003 (*p* = 0.009496), *Incertae Sedis* (*p* = 0.014531), and *Marvinbryantia* (*p* = 0.045719). In contrast, the relative abundance of *Flavonifractor* significantly increased in the treatment group (*p* = 0.029085). These results suggest that *L. paracasei* TISTR 2593 supplementation leads to a significant decrease in the relative abundance of several bacterial taxa in the gut microbiome, except for *Flavonifractor*, which increased significantly. This indicates a selective modulation of the gut microbiome by the probiotic, potentially enhancing the growth of specific beneficial taxa while reducing others.

After 90 days of intervention, a comparison of blood parameters between the placebo and probiotics groups revealed significant differences (Table 1). Notably, the probiotic group exhibited a significant reduction in low-density lipoprotein cholesterol (LDL-C) levels (*p* = 0.027), suggesting a beneficial effect of *L. paracasei* TISTR 2593 supplementation on lipid metabolism. Although other parameters, such as total cholesterol (TC), triglycerides (TG), high-density lipoprotein cholesterol (HDL-C), fasting plasma glucose, TC:HDL-C ratio, LDL-C:HDL-C ratio, and the atherosclerosis index showed no statistically significant differences, trends towards improved lipid profiles and glucose regulation were observed in the probiotic group. These findings underscore the potential of *L. paracasei* TISTR 2593 as a therapeutic adjunct for managing dyslipidemia and associated metabolic disorders, warranting further investigation with larger cohorts and extended follow-up periods to validate these preliminary results.

The analysis of changes in blood parameters from baseline to the end of the study period (Table 2) revealed significant alterations in lipid profiles and glucose levels between the placebo and probiotic groups. Specifically, the probiotic group exhibited a notable reduction in low-density lipoprotein cholesterol (LDL-C) levels (*p* = 0.038), highlighting the lipid-lowering potential of *L. paracasei* TISTR 2593 supplementation. Conversely, the placebo group showed a significant increase in fasting plasma glucose levels (*p* = 0.014), which was not observed in the probiotic group. Other blood parameters, including total cholesterol (TC), triglycerides (TG), high-density lipoprotein cholesterol (HDL-C), TC: HDL-C ratio, LDL-C: HDL-C ratio, and the atherosclerosis index, did not demonstrate significant changes within or between groups. These findings suggest that while *L. paracasei* TISTR 2593 may effectively lower LDL-C, its impact on other metabolic parameters warrants further investigation, especially concerning its potential role in glucose regulation and overall cardiovascular risk profile.

## 4. Discussion

This study evaluated the impact of *L. paracasei* TISTR 2593 supplementation on the gut microbiome of patients with hypercholesterolemia disease through a randomized controlled trial. Our findings demonstrate significant modulation of the gut microbiome composition and increased microbial diversity in the treatment group, suggesting the potential therapeutic benefits of this probiotic strain in managing hypercholesterolemia.

The baseline characteristics of the study participants, as outlined in Tables S1 and S2, reveal a well-matched study population between the placebo and probiotic groups. Table S1 presents a comprehensive comparison of demographic, anthropometric, and lifestyle characteristics at baseline, demonstrating no significant differences between the groups. This includes parameters such as gender distribution, age, body mass index (BMI), body fat percentage, basal metabolic rate, smoking status, alcohol use, and exercise frequency. The lack of significant differences in these baseline characteristics ensures that any observed effects during the intervention can be attributed to the treatment rather than pre-existing disparities between the groups. Table S2 provides an evaluation of blood parameters at baseline, further confirming the comparability of the two groups. Key blood parameters, including total cholesterol (TC), triglycerides (TG), high-density lipoprotein cholesterol (HDL-C), low-density lipoprotein cholesterol (LDL-C), fasting plasma glucose, TC ratio, LDL-C ratio, and the atherosclerosis index, showed no significant differences between the placebo and probiotic groups at the start of the study. This baseline similarity is crucial for the validity of the study’s findings, as it ensures that both groups started from an equivalent point, thereby allowing for a clearer interpretation of the intervention’s effects. The stability of the Shannon diversity index in the placebo group further supports the conclusion that the observed changes in the treatment group are attributable to the probiotic intervention.

The reductions in LDL-C observed in our study suggest the potential benefits of *L. paracasei* TISTR 2593 in managing hypercholesterolemia and reducing cardiovascular risk.

Regarding the mechanism, it is noteworthy that in our earlier study, we observed a significant increase in APOE levels in participants who received *L. paracasei* TISTR 2593 supplementation. APOE plays a crucial role in the clearance of VLDL and LDL from the plasma, which could help explain the reduction in LDL-C levels observed in our current study. The increase in APOE may suggest that *L. paracasei* TISTR 2593 enhances lipid metabolism, contributing to its cholesterol-lowering effects [13]. However, while this provides a potential mechanism, the overall mechanisms by which *L. paracasei* TISTR 2593 exerts these effects are not fully understood. Further studies are needed to explore these biological processes in more detail and to confirm the clinical impact of these findings.

Our study demonstrated a significant reduction in the abundance of *Subdoligranulum* in the probiotic intervention group, highlighting the beneficial impact of *L. paracasei* TISTR 2593 on gut microbiota. The pathogenic potential of *Subdoligranulum* has been implicated in the development of rheumatoid arthritis (RA). Recent research identified a specific *Subdoligranulum* strain associated with autoantibody development in individuals at risk for RA. In a murine model, colonization with this strain led to arthritis with pathology similar to human RA, suggesting that *Subdoligranulum* may drive systemic autoimmunity and joint inflammation through mucosal immune responses [17,18].

By reducing *Subdoligranulum*, our probiotic intervention potentially mitigates the risk of developing RA and other inflammatory conditions. This underscores the therapeutic potential of *L. paracasei* TISTR 2593 in promoting gut health and preventing inflammation-related autoimmune diseases. These findings warrant further investigation to elucidate the underlying mechanisms and long-term benefits of *L. paracasei* TISTR 2593 supplementation in diverse populations.

A recent study suggests a potential negative effect of the *Eubacterium ventriosum* group on scoliosis development. Using a two-sample Mendelian randomization (MR) approach, the study found that the presence of the *Eubacterium ventriosum* group was associated with an increased risk of scoliosis. This further highlights the positive implications of our probiotic intervention, as reducing harmful bacteria like *Subdoligranulum* and potentially detrimental groups like *Eubacterium ventriosum* supports the overall benefits of *L. paracasei* TISTR 2593 supplementation in improving gut health and mitigating associated health risks [19].

The observed increase in *Flavonifractor* abundance in the treatment group is particularly noteworthy. A previous study demonstrated that oral administration of *Flavonifractor* in a mouse model of colitis significantly reduced colonic inflammation through the suppression of IL-17 signaling. This anti-inflammatory effect was attributed to the lipoteichoic acid produced by *Flavonifractor*, highlighting its potential as a therapeutic agent for gut inflammation [20]. The observed increase in *Flavonifractor* in our study suggests that *L. paracasei* TISTR 2593 supplementation may promote a beneficial gut microbiome profile that supports anti-inflammatory processes and gut health. The rise in *Flavonifractor* abundance aligns with its known effects in modulating gut inflammation, thereby enhancing the overall efficacy of our probiotic intervention. This dual impact in reducing harmful bacteria and increasing beneficial ones underscores the therapeutic potential of *L. paracasei* TISTR 2593 in managing gut-related health issues and promoting metabolic well-being.

This selective modulation of gut microbiome composition by *L. paracasei* TISTR 2593 not only enhances the growth of beneficial taxa but also significantly decreases the abundance of other bacterial groups such as *Lachnospiraceae* NK4A136, *Eubacterium ventriosum*, *Phascolarctobacterium* UCG-003, *Incertae Sedis*, and *Marvinbryantia*. The reduction in these taxa suggests a complex interaction within the gut microbiome that requires further investigation. For instance, *Lachnospiraceae* NK4A136 [21] and *Marvinbryantia* [22] are known for short-chain fatty acid (SCFA) production, which plays a vital role in gut health. Changes in these bacteria could impact SCFA levels and overall gut health. The implications of decreased *Eubacterium ventriosum*, *Phascolarctobacterium* UCG-003, and *Incertae Sedis* are less understood, highlighting the need for more in-depth studies to elucidate their roles and the effects of their reduction on host health. This complex modulation underscores the probiotic’s potential to selectively influence the gut microbiome, promoting beneficial changes while reducing potentially harmful bacteria. Further research is warranted to explore the long-term benefits and specific mechanisms by which this probiotic strain influences gut health.

One of the strengths of this study is the randomized controlled design, which minimizes bias and enhances the reliability of the results. The comprehensive analysis of gut microbiome composition using advanced sequencing techniques provides robust data on microbial changes. However, the study has several limitations. First, as a pilot study, the sample size was relatively small, which may limit the generalizability of the findings and the ability to detect more subtle effects. Second, while we observed significant changes in certain bacterial taxa, the overall diversity of the gut microbiome did not differ significantly between the treatment and placebo groups, suggesting that the effects of *L. paracasei* TISTR 2593 may be specific to certain bacteria rather than broad-spectrum. Third, the study did not include a detailed dietary assessment, which could have influenced gut microbiome composition and metabolic outcomes. Additionally, the short duration of the intervention may not have been sufficient to observe long-term effects on metabolic health or cardiovascular risk markers. Lastly, the mechanisms underlying the observed changes in LDL-C and specific gut bacteria remain unclear, highlighting the need for further research, including larger, longer-term studies with more comprehensive metabolic and microbiome analyses, such as metabolomics.

## 5. Conclusions

In conclusion, *L. paracasei* TISTR 2593 supplementation may influence gut microbiome composition in patients with hypercholesterolemia. These changes could be associated with potential improvements in metabolic health, although further research is needed to confirm these findings. Future studies should aim to elucidate the specific metabolic pathways affected by this probiotic and to explore its long-term effects on gut health and metabolic outcomes. Additionally, expanding research to include diverse populations and varying metabolic conditions will be important in determining the broader applicability of *L. paracasei* TISTR 2593 as a potential therapeutic intervention.

## Figures and Tables

**Table 1:** Comparison of blood parameters at 90 days [13].

Blood Parameter	Placebo	Probiotics	*p*-Value
TC (mg/dL)	246.70 ± 40.42	227.75 ± 33.45	0.876
TG (mg/dL)	157.14 ± 66.99	151.21 ± 41.42	0.115
HDL-C (mg/dL)	57.00 ± 10.90	55.85 ± 10.83	0.870
LDL-C (mg/dL)	163.00 ± 39.94	137.63 ± 29.24	0.027
Fasting plasma glucose (mg/dL)	101.50 ± 22.61	96.70 ± 8.40	0.063
TC: HDL-C	3.10 ± 1.57	2.48 ± 1.15	0.062
LDL-C: HDL-C	2.68 ± 1.30	2.44 ± 0.59	0.652
Atherosclerosis index	250.47 ± 40.52	225.38 ± 33.23	0.262

Data presented as mean ± standard deviations. *p*-value was calculated by *t*-test to indicate inter-group difference. TC: total cholesterol, TG: triglyceride, HDL-C: high-density lipoprotein cholesterol, LDL-C: low-density lipoprotein cholesterol.

**Table 2:** Changes in blood parameters pre- and post-study [13].

Blood Parameter	Placebo				Probiotics			
	Mean Difference	95% CI		*p*-Value	Mean Difference	95% CI		*p*-Value
TC (mg/dL)	15.22	−3.20	42.50	0.304	−5.75	−18.06	22.64	0.565
TG (mg/dL)	11.06	−10.73	30.43	0.563	7.71	−21.53	22.71	0.496
HDL-C (mg/dL)	4.52	−3.52	13.42	0.357	2.50	−2.37	12.49	0.342
LDL-C (mg/dL)	11.39	−5.29	35.09	0.532	−17.52	−30.32	2.08	0.038
Fasting plasma glucose (mg/dL)	5.41	2.27	10.83	0.014	0.88	−2.84	4.60	0.492
TC: HDL-C	0.20	−1.02	1.22	0.785	−0.46	−1.59	0.11	0.712
LDL-C: HDL-C	−0.28	−1.44	0.84	0.446	−0.53	−1.78	1.32	0.361
Atherosclerosis index	19.35	2.26	46.21	0.293	−7.77	−24.68	18.87	0.484

*p*-value was calculated by paired *t*-test to indicate intra-group difference. CI: confidence interval, TC: total cholesterol, TG: triglyceride, HDL-C: high-density lipoprotein cholesterol, LDL-C: low-density lipoprotein cholesterol.

## Data Availability

The data supporting the reported results of this study are available upon request due to ethical restrictions. Researchers interested in accessing the data can contact the corresponding author.

## Supplementary Materials

The following supporting information can be downloaded at https://www.mdpi.com/article/10.3390/nu16172916/s1. Figure S1: Orthogonal Partial Least Squares Discriminant Analysis (OPLS-DA) using 200 bacterial genera differentiates between placebo and probiotic groups; Table S1: Comparison of demographic, anthropometric and lifestyle characteristics at baseline; Table S2: Evaluation of blood parameters in both groups at baseline; Table S3: VIP scores from OPLS-DA analysis.
