# Supplementary material for: Impact of Lactocaseibacillus (Lactobacillus) paracasei sup. paracasei TISTR 2593 Probiotic Supplementation on the Gut Microbiome of Hypercholesterolemia Patients: A Randomized Controlled Trial
Source: Nutrients. 2024 Sep 1;16(17):2916. doi: 10.3390/nu16172916 (PMC11397238; doi:10.3390/nu16172916)
Supplement: Supplementary file 1 [file nutrients-16-02916-s001.zip › nutrients-3147171-supplementary.pdf]

Supplementary data

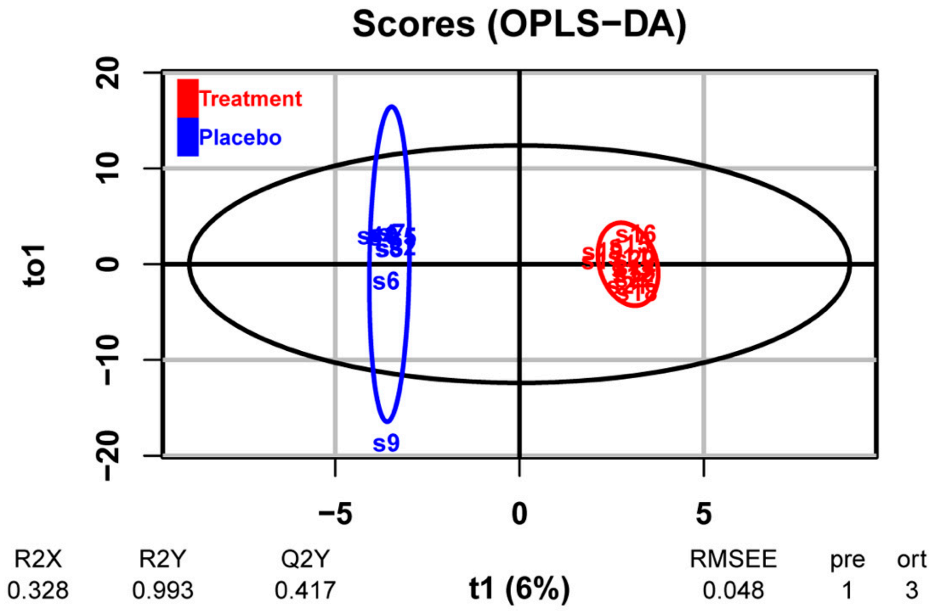

Figure S1: Orthogonal Partial Least Squares Discriminant Analysis (OPLS-DA) using 200 bacterial genera differentiates between placebo and probiotic groups

Table S1: Comparison of demographic, anthropometric and lifestyle characteristics at baseline

Characteristics	Placebo group (n = 21)		Probiotic group (n = 21)		*p*-value
	%	Mean±SD	%	Mean±SD	
**Gender**					0.179[c]
Male	65.22	N/A	82.61	N/A	
Female	34.78	N/A	17.39	N/A	
Age (year)	N/A	48.50 ± 5.30	N/A	46.00 ± 5.10	0.655[t]
BMI (kg/m2)	N/A	25.11 ± 4.24	N/A	26.21 ± 3.82	0.384[t]
Body Fat percentage (%)	N/A	30.84 ± 7.30	N/A	34.30 ± 7.37	0.489[t]
Basal Metabolism Rate (Kcal)	N/A	1354.78 ± 276.48	N/A	1232.22 ± 218.93	0.282[t]
**Smoke**					N/A
Yes	0.0	N/A	0.0	N/A	
No	100	N/A	100	N/A	
**Alcohol use**					0.432[c]
Never	85.71	N/A	76.19	N/A	
Ever (2-3 times/week)	14.29	N/A	23.81	N/A	
More than 2-3 times/week	0.0	N/A	0.0	0.0	
**Exercise**					0.659[c]
Never	14.29	N/A	23.810	N/A	
Ever (2-3 times/week)	52.38	N/A	47.619	N/A	
More than 2-3 times/week	33.33	N/A	28.571	0.0	

N/A; Not applicable c; calculated by the Chi-square test, indicates difference between the placebo and probiotic groups. t; calculated by the t-test, indicates difference between the placebo and probiotic groups.

Table S2: Evaluation of blood parameters in both groups at baseline

Blood parameters	Placebo group	Probiotic group	*p*-value
TC (mg/dL)	231.48 ± 40.51	233.50 ± 41.59	0.635
TG (mg/dL)	146.09 ± 55.74	143.50 ± 40.05	0.627
HDL-C (mg/dL)	52.48 ± 10.65	53.35 ± 10.71	0.942
LDL-C (mg/dL)	151.61 ± 36.13	155.15 ± 33.03	0.472
Fasing plasma glucose (mg/dL)	96.09 ± 15.78	95.82 ± 8.13	0.234
TC:HDL-C	2.90 ± 1.51	2.94 ± 1.35	0.198
LDL-C:HDL-C	2.96 ± 0.92	2.97 ± 0.66	0.845
Artherosclerosis index	231.12 ± 40.59	233.15 ± 41.62	0.871

p-value was calculated by t-test to indicate inter-group difference at the baseline and end of the intervention period. TC; Total cholesterol, TG; Triglyceride, HDL-C; High-Density Lipoprotein Cholesterol, LDL-C; Low-Density Lipoprotein Cholesterol.

Table S3: VIP scores from OPLS-DA analysis

Genus	VIP>1
p__Firmicutes.f__Lachnospiraceae.g__Blautia	1.05111187
p__Firmicutes.f__Lachnospiraceae.g__.Ruminococcus._torques_group	1.62652
p__Firmicutes.f__Lachnospiraceae.g__Agathobacter	1.06789009
p__Firmicutes.f__Ruminococcaceae.g__Subdoligranulum	1.53083755
p__Firmicutes.f__Erysipelotrichaceae.g__Holdemanella	1.20460651
p__Fusobacteriota.f__Fusobacteriaceae.g__Fusobacterium	1.26937386
p__Firmicutes.f__Lachnospiraceae.g__.Ruminococcus._gauvreauii_group	1.55846886
p__Firmicutes.f__Lachnospiraceae.g__.Eubacterium._hallii_group	1.76764572
p__Firmicutes.f__Ruminococcaceae.g__Ruminococcus	1.00259693
p__Firmicutes.f__Lachnospiraceae.g__.Eubacterium._eligens_group	1.09576614
p__Proteobacteria.f__Enterobacteriaceae.g__Escherichia.Shigella	1.06677639
p__Firmicutes.f__Oscillospiraceae.g__UCG.002	1.41781807
p__Firmicutes.f__Ruminococcaceae.g__CAG.352	1.7691794
p__Firmicutes.f__.Eubacterium._coprostanoligenes_group. g__.Eubacterium._coprostanoligenes_group	2.09777553
p__Firmicutes.f__Lachnospiraceae.g__Lachnospiraceae_NK4A136_group	1.90447222
p__Firmicutes.f__Ruminococcaceae	1.17388358
p__Bacteroidota.f__Muribaculaceae.g__Muribaculaceae	1.56026112
p__Firmicutes.f__Lachnospiraceae.g__.Ruminococcus._gnavus_group	1.38252203
p__Firmicutes.f__Lachnospiraceae.g__.Eubacterium._ventriosum_group	1.65486638
p__Firmicutes.f__Monoglobaceae.g__Monoglobus	1.58281488
p__Firmicutes.f__Acidaminococcaceae.g__Phascolarctobacterium	1.9989528
p__Firmicutes.f__Veillonellaceae.g__Dialister	1.70806801
p__Firmicutes.f__Oscillospiraceae.g__UCG.003	2.5173261
p__Firmicutes.f__Leuconostocaceae.g__Weissella	1.22783371
p__Firmicutes.f__Christensenellaceae.g__Christensenellaceae_R.7_group	1.4135447
p__Firmicutes.f__Lachnospiraceae.g__Lachnospiraceae_UCG.001	1.02949762
p__Firmicutes.f__Ruminococcaceae.g__.Eubacterium._siraeum_group	1.12728409
p__Firmicutes.f__Ruminococcaceae.g__Incertae_Sedis	1.9096486
p__Firmicutes.f__Lachnospiraceae.g__Marvinbryantia	1.5666686
p__Firmicutes.f__Lachnospiraceae.g__Lachnospiraceae_FCS020_group	1.94756196
p__Firmicutes.f__Oscillospiraceae.g__NK4A214_group	1.36175425
p__Firmicutes.f__Lactobacillaceae.g__Lactobacillus	1.20856192
p__Firmicutes.f__Lachnospiraceae.g__.Eubacterium._xylanophilum_group	1.34371807
p__Firmicutes.f__UCG.010.g__UCG.010	1.06487858
p__Firmicutes.f__Ruminococcaceae.g__uncultured	1.27691618
p__Proteobacteria.f__Enterobacteriaceae.g__Enterobacter	1.34486263
p__Actinobacteriota.f__Eggerthellaceae.g__Senegalimassilia	1.38742428

p__Firmicutes.f__Oscillospiraceae.g__Colidextribacter	1.33301559
p__Firmicutes.f__Anaerovoracaceae.g__Family_XIII_AD3011_group	1.18971209
p__Firmicutes.f__Streptococcaceae.g__Lactococcus	1.23669133
p__Firmicutes.f__Oscillospiraceae.g__Flavonifractor	1.89871801
p__Firmicutes.f__Enterococcaceae.g__Enterococcus	1.58147979
p__Bacteroidota.f__Prevotellaceae.g__Prevotellaceae_NK3B31_group	1.16785013
p__Desulfobacterota.f__Desulfovibrionaceae.g__Bilophila	1.09781305
p__Proteobacteria.f__uncultured.g__uncultured	1.26959483
p__Firmicutes.f__Peptostreptococcales.Tissierellales.g__Finegoldia	1.47610599
p__Proteobacteria.f__Enterobacteriaceae.g__Plesiomonas	1.03335682
p__Firmicutes.f__Erysipelatoclostridiaceae.g__Erysipelatoclostridium	1.08640607
p__Firmicutes.f__Peptostreptococcales.Tissierellales.g__Fenollaria	1.55531875
p__Firmicutes.f__Peptostreptococcales.Tissierellales.g__Peptoniphilus	1.5217355
p__Firmicutes.f__Anaerovoracaceae.g__Family_XIII_UCG.001	1.10044157
p__Bacteroidota.f__Prevotellaceae.g__Prevotellaceae_UCG.003	1.00178244
p__Firmicutes.f__Veillonellaceae.g__Veillonella	1.3339132
p__Firmicutes.f__Leuconostocaceae.g__Leuconostoc	1.42698268
p__Actinobacteriota.f__Micrococcaceae.g__Rothia	1.65145728
p__Firmicutes.f__Peptostreptococcales.Tissierellales.g__Anaerococcus	1.43521716
p__Firmicutes.f__Veillonellaceae.g__Allisonella	1.21575132
p__Thermoplasmatota.f__Methanomethylophilaceae.g__Candidatus_Methanomethylophilus	1.00178244
p__Firmicutes.f__Erysipelotrichaceae.g__Holdemania	1.23607376
p__Firmicutes.f__Peptostreptococcales.Tissierellales.g__Murdochiella	1.33881213
p__Euryarchaeota.f__Methanobacteriaceae.g__Methanosphaera	1.00370146
p__Firmicutes.f__Peptostreptococcales.Tissierellales.g__Ezakiella	1.62816681
p__Campilobacterota.f__Campylobacteraceae.g__Campylobacter	1.23598646
p__Bacteroidota.f__uncultured.g__uncultured	1.03335682
p__Firmicutes.f__Erysipelotrichaceae.g__Erysipelotrichaceae_UCG.006	1.07881995
p__Firmicutes.f__RF39.g__RF39	1.00178244
p__Bacteroidota.f__Porphyromonadaceae.g__Porphyromonas	1.23306657
p__Verrucomicrobiota.f__vadinBE97.g__vadinBE97	1.02984972
p__Firmicutes.f__Ruminococcaceae.g__Paludicola	1.00370146
p__Firmicutes.f__Lachnospiraceae.g__Lachnospiraceae	1.06751856
p__Actinobacteriota.f__Actinomycetaceae.g__Actinomyces	1.23725198
p__Firmicutes.o__Oscillospirales	1.00370146
p__Firmicutes.f__Erysipelatoclostridiaceae.g__UCG.004	1.00178244
p__Firmicutes.f__Peptococcaceae.g__uncultured	1.15666996
